# Numerical Study Using Microstructure Based Finite Element Modeling of the Onset of Convective Heat Transfer in Closed-Cell Polymeric Foam

**DOI:** 10.3390/polym13111769

**Published:** 2021-05-28

**Authors:** Jorge-Enrique Rivera-Salinas, Karla-Monzerratt Gregorio-Jáuregui, Heidi-Andrea Fonseca-Florido, Carlos-Alberto Ávila-Orta, Eduardo Ramírez-Vargas, José-Antonio Romero-Serrano, Alejandro Cruz-Ramírez, Víctor-Hugo Gutierréz-Pérez, Seydy-Lizbeth Olvera-Vazquez, Lucero Rosales-Marines

**Affiliations:** 1 Catedrático CONACyT—Departamento de Procesos de transformación, Centro de Investigación en Química Aplicada—CIQA, Saltillo 25294, Coahuila, Mexico; 2 Departamento de Ingeniería en Metalurgia y Materiales, Instituto Politécnico Nacional, Escuela Superior de Ingeniería Química e Industrias Extractivas—ESIQIE, UPALM, Ciudad de México 07738, CDMX, Mexico; kaarlaa@gmail.com (K.-M.G.-J.); romeroipn@hotmail.com (J.-A.R.-S.); alcruzr@ipn.mx (A.C.-R.); 3 Catedrático CONACyT—Departamento de Materiales Avanzados, Centro de Investigación en Química Aplicada—CIQA, Saltillo 25294, Coahuila, Mexico; heidi.fonseca@ciqa.edu.mx; 4 Departamento de Materiales Avanzados, Centro de Investigación en Química Aplicada—CIQA, Saltillo 25294, Coahuila, Mexico; carlos.avila@ciqa.edu.mx; 5 Departamento de Procesos de transformación, Centro de Investigación en Química Aplicada—CIQA, Saltillo 25294, Coahuila, Mexico; eduardo.ramirez@ciqa.edu.mx; 6 Departamento de Formación Profesional Específica, Instituto Politécnico Nacional—Unidad Profesional Interdisciplinaria de Ingeniería Campus Zacatecas—UPIIZ, Zacatecas 98160, Zacatecas, Mexico; metalurgico2@hotmail.com (V.-H.G.-P.); seydyliz@hotmail.com (S.-L.O.-V.); 7 Facultad de Ciencias Químicas, Universidad Autónoma de Coahuila, Blvd. Venustiano Carranza e Ing. José Cárdenas Valdés, Saltillo 2528, Coahuila, Mexico; lucero_rosales@uadec.edu.mx

**Keywords:** thermal conductivity, closed-cell foams, convective heat transport, finite element analysis

## Abstract

The thermal performance of closed-cell foams as an insulation device depends on the thermal conductivity. In these systems, the heat transfer mode associated with the convective contribution is generally ignored, and studies are based on the thermo-physical properties that emerge from the conductive contribution, while others include a term for radiative transport. The criterion found in the literature for disregarding convective heat flux is the cell diameter; however, the cell size for which convection is effectively suppressed has not been clearly disclosed, and it is variously quoted in the range 3–10 mm. In practice, changes in thermal conductivity are also attributed to the convection heat transfer mode; hence, natural convection in porous materials is worthy of research. This work extends the field of study of conjugate heat transfer (convection and conduction) in cellular materials using microstructure-based finite element analysis. For air-based insulating materials, the criteria to consider natural convection ($Ra=10^{3}$) is met by cavities with sizes of 9.06 mm; however, convection is developed into several cavities despite their sizes being lower than 9.06 mm, hence, the average pore size that can effectively suppress the convective heat transfer is 6.0 mm. The amount of heat transported by convection is about 20% of the heat transported by conduction within the foam in a $Ra=10^{3}$, which, in turn, produces an increasing average of the conductivity of about 4.5%, with respect to a constant value.

## 1. Introduction

Closed-cell foams are widely used as thermal insulators, and according to the intended application, the solid matrix could be made of polymers, metals, and ceramics. For applications requiring lightweight and high-insulation efficiencies, Polyvinyl Chloride (PVC), expanded polystyrene (EPS), polyethylene (PE), and ethylene vinyl acetate (EVA) are typically used insulation materials [1]. Polymeric cellular materials have porosity >95%. For applications requiring lightweight structural, energy absorption, and thermal insulation devices, aluminum metal foams are typically used, whose porosity ranges between 0.8 and 0.95 [2]. For applications where thermal-shock resistance is a requisite, porous ceramics are ideal candidates [3]. The thermal performance of cellular material as an insulation device depends on the magnitude of the effective thermal conductivity ($\kappa_{e}$) as well as its capacity to be unaffected by factors such as the operative temperature. Low thermal conductivity in cellular matrixes takes advantage of the gases’ low conductivity, occluded in the cavities. Materials with low densities pose a very high fraction of the gaseous phase in the cellular matrix.

For the sustained interest in the development of low thermal conductivity materials, diverse models have been developed, which enable the determination of the effective thermal conductivity for porous materials during the thermal design. These models are classified into three broad categories based on the following approaches: (i) analytical models derived by solving Laplace’s heat conduction equation, (ii) empirical-correlations-based models, and (iii) numerical-simulation-based models [4].

Although analytical models are straightforward approaches to material design, they should be fed with topological information obtained from advanced foam modeling techniques to increase their accuracy. In addition, to make the model mathematically tractable, simplifying hypotheses for the microstructure and physics. A complete description of the structure is generated by means of X-ray micro-computed microtomography (μ−CT) imaging.

Empirical correlations are formulated based on empirical parameters by fitting experimental data; therefore, its accuracy depends on the calibration of the available experimental measurements. A review of some of the analytical and empirical correlations proposed in the literature to predict the thermal conductivity have shown that, for a gas-filled polymer, none of the tested theoretical models have proved adequate [5]. Numerical investigation of conductive heat transfer in high-porosity foams estimated the degree of reliability and the domain of applicability for a large number of analytical and empirical expressions suggested for predicting the thermal conductivity of high-porosity foams [6].

In recent decades, mathematical models to investigate the heat transfer behavior of closed-cell foams have been solved numerically through different discrete element methods, such as finite element method (FEM), and finite volume method (FVM). A literature review of traditional and advanced techniques to predict the thermal conductivity of foamed materials showed that numerical analysis is an accurate approach to this end [7,8,9]. In the numerical analysis of thermal transport, the foam structure can be considered as a simple model that features a regular array of circles to produce closed-cell foam structures. On the other hand, the structure can incorporate stochastic features in the cell if the models are generated by Voronoi tessellation and Laguerre tessellation. The advantages and disadvantages of modeling thermal transport using the Voronoi tessellation and Laguerre tessellation are discussed in [9]. In that work, the authors also introduced a new modelling method to generate the closed-cell foam models which consider the various cell wall thickness distributions, and different cell shapes. It is important to remark that the results obtained in that research show that for a given relative density, the thermal conductivity of closed-cell foams reduces as the cell size and shape anisotropy increases. However, the study was limited to a maximum cell size of 4.08 mm. On the other hand, for a realistic representation, and more precise description, the foam structure can be recovered after a μ−CT scan to study a 3D volume description of the solid volume as input, or 2D cellular microstructure from a slice of μ−CT [6].

Regardless of the method used to construct the microstructure of the closed-cell foam for numerical analysis, typically, the finite element analysis (FEA) relies on the concept of the representative elementary volume (REV) of the material, which comprises a predetermined number of cells, voids, or inclusions to relate the heterogeneous material to the homogeneous medium using computational homogenization [10]. The REV represents the smallest volume of material that describes the global characteristics of the material [10]. Although finite element analysis is computationally expensive, it may account for the thermal behavior concerning the actual microstructure, i.e., randomly oriented cells, and the inhomogeneity in the size and shape of cells or cavities. As a result, it represents an enhanced description that enables the detection of salient features of thermal transport as well as various transport phenomena, which allows for better thermal solutions to be established.

The energy transfer within the bulk cellular materials is carry out via three competing mechanisms: conduction across the material matrix and the occluded gas, natural convection of the occluded gas in the pores, and radiation within the internal solid surfaces of the matrix. The effective or overall thermal conductivity is depicted as the result of these additive terms, where the conductive heat transfer is the most dominant; therefore, the transport phenomena in such systems are mainly described by the thermo-physical properties that emerge from the conductive transport [4,5,6,11,12,13]. Some descriptions have considered a term for radiative heat transfer flux, and they suggest that radiation may contribute 6–26% of the effective conductivity [5]. Very detailed numerical investigations of the radiative transfer on several polymeric foams were performed in the literature to study the radiative properties of the foams as a function of cell size and wall geometry [14,15,16]. In most cases, convective heat transfer is not considered by arguing that the associated heat transfer caused by this is insignificant for cell diameters less than 4 mm [5,11,13,17]. The investigation into the heat transfer rate due to natural convection within the microstructures of closed-cell cellular materials has received much less attention than the conductive and radiative heat transfer in the experimental and numerical studies of thermal transport [2]. Therefore, a comprehensive quantitative description of this heat transfer mode to bulk conductivity in cellular materials is missing in the literature. In that sense, there are still controversies in the literature regarding the role of convection contribution in the overall heat transfer in cellular materials. For example, the criterion used to disregard the convective effects on the thermal conductivity is the cell diameter; however, for air-filled foams which are widely used in practical applications, the cell diameter is variously quoted in the range 3–10 mm [5,6,11,13,17,18,19]. As there is limited information on the heat transfer rate promoted by convection as a function of the cell size in the micro-scale, the cavity size for which convection is effectively suppressed has not been disclosed. Experimental results in air-filled foam show that a 4 mm diameter corresponds to a critical Rayleigh number ($Ra=\frac{g\alpha d^{3}\Delta T}{kv}$) of 50. Appreciable convective effects correspond to a Rayleigh number of 10^3^. Though, other authors have indicated an opposite scenario by arguing that convection effects could still be significant in cell diameters smaller than 3 mm, or even in the 1.5 mm diameter region in the case of chlorofluorocarbon-filled foams [11]. Progelhof et al. [5] argue that convective heat transfer in foams depends not only on the cell diameter but also on the gas properties. The authors also point out that because the cavities of the cellular materials are not true spheres, but ellipsoidal in shape and randomly oriented, convective effects in the direction of the major axis of the cell are greater than that in the direction of the minor axis and, therefore, the convective transport exhibits an anisotropic behavior in foam. On the other hand, entropy generation in cavities is receiving considerable attention as it indicates the efficiency of a power/refrigeration systems according to the second law of thermodynamics. In this context, free convective heat transfer, including the second law of thermodynamics in microfluids within a rectangular enclosure at different angles of inclination, have been numerically analyzed. It was found that upon increasing the Rayleigh number, the heat transfer rate and total entropy generation increase, and, as the fluid becomes more conductive (increasing the nanoparticle volume fraction), the heat transfer rate decreases [18].

H. Jeffreys [19] introduced a dimensionless parameter (λ) that accounts for the onset of convective flow in a layer of incompressible fluid when the temperature decreases upwards. The expression is given by:

(1) $$\lambda =\frac{g\alpha \beta d^{3}}{\kappa \nu }>1709$$

where g is gravity, α the coefficient of expansion of the fluid, β the gradient of temperature across the cells, d the diameter of the cell, κ thermal diffusivity and,ν is kinematic viscosity. The Rayleigh number (Ra) and Jeffreys parameter (λ) are essentially the same definitions. Rayleigh derived a solution of the problem by adopting boundary conditions that correspond to a fluid with a free surface at the top and bottom and constant temperature over both, whereas Jeffreys adopted non-slip boundary conditions at the top and bottom [19]. These expressions agreed regarding the fact that convective motion is produced by a specific vertical temperature gradient across the fluid, whereas viscosity and thermal conduction may delay the onset of convective motion. These dimensionless parameters Ra and λ, represent the ratio of the destabilizing effect of buoyancy (so that the fluid begins to move) to the viscous damping (the hindered motion of the fluid by the viscosity).

On the other hand, in the case of open-celled metal foams, the effect of natural convection in the effective thermal conductivity of the foam was exposed by comparing the results, measured under atmospheric and vacuum conditions. The effective thermal conductivity at ambient pressure was twice the value of vacuum condition for a given temperature, which was somewhat unexpected, as it was estimated that the contribution due to natural convection was negligible because the cell sizes of the foam were < 5 mm; however, as the metal foams were highly porous (>0.9) and the open cells were all interconnected, it was concluded that natural convection took place in a global domain, rather than being limited to a single cell [20]. Current research concerning the study of the performance of high-porosity polymer foams as insulating layers reports that variations in the thermal conductivity with transient temperature are more pronounced for materials with low densities, given their larger air volume ratio. These variations are attributed to the higher importance of convective and radiative heat transfer mechanism within the porous material [21]. The cell diameter is expected to be larger at a higher porosity, which can activate gaseous convection [5].

The literature survey also shows that it is worth extending the field of study of the natural convection heat transfer mode in cellular materials to investigate the correlation between variations in the thermal conductivity value, and the rate of heat transfer activated by convection. The role of convective contribution in the thermal conductivity of closed-cell porous media has never been numerically investigated using real microstructures. Determination of the maximum cell size which effectively suppresses the convection of the gas occluded in the cavity, including randomly oriented cavities of different shapes, and wall thickness variations, can be tackled using a microstructure-based finite element analysis of foam. Finite element modelling of the actual structure of cellular materials allows for careful study of the impact of specific parameters, such as cell size and cavity shapes, on the convective currents, and their relationship with the macroscopic thermal response of closed-cell foams. The purpose of the current research is to determine the cell size that effectively suppresses convection in air-filled foams, providing a quantitative description of the convective heat transfer in these systems, and its impact on thermal conductivity value (macroscopic response) as a function of the average pore size. A two-dimensional time-dependent numerical model was developed to determine laminar-free convection using a microstructure-based finite element analysis of a polymeric foam. The onset of convective heat transfer corresponds to a pore size of 9.06 mm and, despite the fact that natural convection took place into a single cell, it has an impact on the surrounding cavities, even for sizes lower than 9.0 mm. Therefore, convection takes place in a global domain, and it should be considered that, for air-based insulating materials, the onset of convective heat transfer corresponds to an average pore size of 6.1 mm. At this pore level, the amount of heat transported by convection is about 20% of the heat transported by conduction within the foam, which produces an increasing average of the thermal conductivity of about 4.5% concerning the constant value.

## 2. Finite Element Analysis of Closed-Cell Foam

### 2.1. Computational Domain

**Figure 1:**
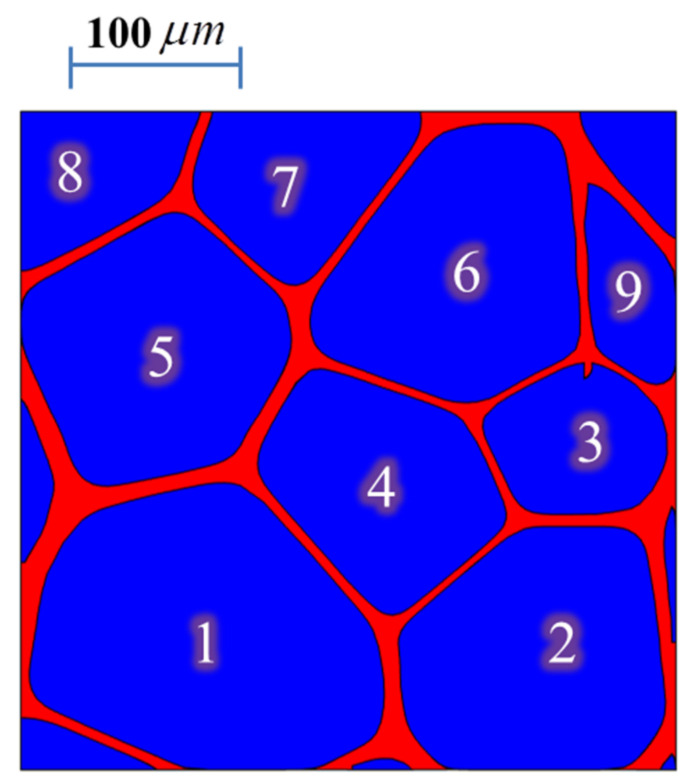
Microstructure of closed-cell PVC foam [6].

As mentioned above, the cell structure found in the closed-cell foams materials poses a large number of cells, randomly distributed, with a wide variety of sizes and shapes, as well as variations in wall thickness. To generate an appropriate foam model, a real porous microstructure of plastic polyvinyl chloride (PVC) closed-cell foam with a solid fraction of 0.196 was used. The foam model is shown in Figure 1. This image comes from a slice of the tomographic 3-D images of PVC obtained by Coquart et al. [6] after thresholding and filtering operations.

Flow visualization has shown that the resulting motion by natural convection of the fluid entrapped in closed cavities is truly two-dimensional [22]; hence, the image shown in Figure 1 is appropriated to incorporate realistic microstructural features of foamed materials in the computational domain and allows for the proper definition of conjugate natural convection with conducting walls. Additionally, in the work of Coquart et al. [6], 3-D images were used to study numerically the conductive behavior of this polymer foam. Hence, the REV constructed using the slice of the tomographic 3-D image fulfills the representative features of the material to study the macroscopic response, i.e., the effective thermal conductivity [6]. The binary image after processing (raster image)—a slice of the tomographic 3-D image—was converted to a vector image (v.dx file) to set the computational domain for the FEA, as was done in [23]. To find the average cell size which suppresses gas convection, the REV size was scaled by 40×, 50×, and 60× in a parametric study to produce a Rayleigh number of 10^3^ in at least one of the cells. In Figure 1, pores are numbered for the individual cell characterization, as listed in Table 1.

### 2.2. Structural Parameters in PVC Microstructure

The microstructure of PVC poses cell sizes inhomogeneity and cell shape irregularity (Figure 1). The structural parameters such as the cell diameters, and shape of the cells were characterized in terms of the equivalent diameter (d) and the shape factor (S) respectively, which are given as follows [24]:

(2) $$d=\frac{4A}{\Pi }$$

(3) $$S=\frac{\Pi^{2}}{4\pi A}$$

Here, A is the area of cell, and Π is the perimeter of the cavities. The equivalent diameter corresponds to the diameter of a circular pore with the same area of the measured cell. The relationship between shape factor and regular pore shape is given in Table 1 [24]. Table 2 gives the equivalent diameter and the shape factor of an individual cell of the as-processed PVC microstructure (Figure 1) to characterize the degree of cell size inhomogeneity and shape irregularity in foam. The surface area ($A_{cell}$) and perimeter ($p_{cell}$) of the isolated cavities were computed using Equations (4) and (5), respectively

(4) $$A_{}=\iint dS$$

(5) $$\Pi =\int_{C}^{}dl$$

where S is a parametric surface of the pores, while C is the pores’ contour.

In Table 2, it can be seen that the maximum cell size ratio found in the PVC foam is about 2.8, whereas the pore or cavity shapes are closer to circular and ellipsoidal (1:2) shapes.

### 2.3. Conjugate Heat Transfer

The set of governing equations for conjugate heat transfer, the combination of conduction heat transfer in a PVC foam structure, and convection heat transfer in the air occluded in the pores for unsteady state, along with their corresponding boundary conditions, are described in this section. The set of partial differential equations (PDE) satisfies the law of conservation of mass, momentum, and energy. The computational procedure used for the solution of the governing equations is then briefly described. The model was implemented in COMSOL Multiphysics.

Energy equation for solid

(6) $$\rho_{PVC}c_{p}{}_{,PVC}\frac{\partial T}{\partial t}=\frac{\partial }{\partial x}\left(k_{PVC}\frac{\partial T}{\partial x}\right)+\frac{\partial }{\partial y}\left(k_{PVC}\frac{\partial T}{\partial y}\right)$$

Energy equation for fluid

(7) $$\rho {\left(T\right)}_{air}c_{p}{\left(T\right)}_{air}\left(\frac{\partial T}{\partial t}+u\frac{\partial T}{\partial x}+v\frac{\partial T}{\partial y}\right)=\frac{\partial }{\partial x}\left(k{\left(T\right)}_{air}\frac{\partial T}{\partial x}\right)+\frac{\partial }{\partial y}\left(k{\left(T\right)}_{air}\frac{\partial T}{\partial y}\right)$$

Momentum equations for fluid

(8) $$\rho {\left(T\right)}_{air}\left(\frac{\partial u}{\partial t}+u\frac{\partial u}{\partial x}+v\frac{\partial u}{\partial y}\right)=-\frac{\partial p}{\partial x}+\mu {\left(T\right)}_{air}\left(\frac{\partial^{2}u}{\partial x^{2}}+\frac{\partial^{2}u}{\partial y^{2}}\right)$$

(9) $$\rho {\left(T\right)}_{air}\left(\frac{\partial v}{\partial t}+u\frac{\partial v}{\partial x}+v\frac{\partial v}{\partial y}\right)=-\frac{\partial p}{\partial y}+\mu {\left(T\right)}_{air}\left(\frac{\partial^{2}v}{\partial x^{2}}+\frac{\partial^{2}v}{\partial y^{2}}\right)+\rho {\left(T\right)}_{air}g$$

Continuity equation for a compressible fluid

(10) $$\frac{\partial \rho {\left(T\right)}_{air}}{\partial t}+\frac{\partial }{\partial x}\left(\rho {\left(T\right)}_{air}u\right)+\frac{\partial }{\partial y}\left(\rho {\left(T\right)}_{air}v\right)=0$$

where $\rho {\left(T\right)}_{air}$ and $\rho_{PVC}$ are the density of air and PVC, respectively. u and v denote the velocity in x and y directions, respectively, p is pressure, T is temperature, $\mu {\left(T\right)}_{air}$ is kinematic viscosity, $k{\left(T\right)}_{air}$ and $k_{PVC}$ are thermal diffusivities, while $c_{p}{\left(T\right)}_{air}$ and $c_{p,PVC}$ are heat capacities. Radiation heat exchange between the walls of the cellular structure is neglected. The boundary conditions include the no-slip on the wall of cavities (Equation (11)), whereas across the air/PVC interface, the temperature and heat flux are continuous (Equations (12) and (13), respectively). For fluid on the outer surfaces of the REV, the boundaries were symmetry boundary conditions (Equation (14)).

(11) u=v=0

(12) $$T_{air}=T_{PVC}$$

(13) $$\alpha {\left(T\right)}_{air}\frac{\partial T}{\partial x}n_{x}+\alpha {\left(T\right)}_{air}\frac{\partial T}{\partial y}n_{y}=\alpha_{PVC}\frac{\partial T}{\partial x}n_{x}+\alpha_{PVC}\frac{\partial T}{\partial y}n_{y}$$

(14) $$un_{x}+vn_{y}=0$$

where $\alpha {\left(T\right)}_{air}$ and $\alpha_{PVC}$ are thermal diffusivities and $n_{x}$ and $n_{y}$ denote the surface normal. The heat was transferred from the bottom to the top with a temperature differential of 27.8 °C across the material as specified by the ASTM C518. The cold side (top boundary) was set to 10 °C and the warm side (bottom boundary) to 37 °C. On the two vertical walls on the outer REV, the boundaries were assumed to be insulated. Air material properties are temperature-dependent, and air density variations are considered in terms of pressure and temperature using the ideal gas law. The reference pressure level is 1 atm. Initially, the fluid is taken to be stagnant. The properties of the solid domain used in the thermal study, such as the density and heat capacity of the matrix correspond to PVC material, with a value of $k_{PVC}=0.16{\;\mathrm{Wm}}^{-1}\;K^{-1}$ [6].

**Figure 2:**
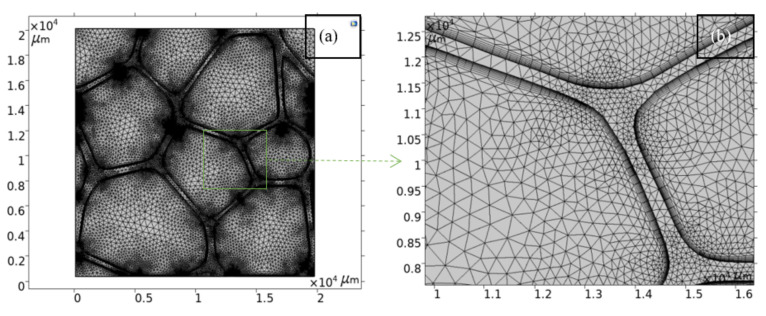
(**a**) Mesh structure used in the foam model of the largest scaling studied, and (**b**) zoom to show the finest mesh at the walls of cavities.

The governing equations are solved using the Galerkin finite element method (FEM). A fully coupled solution for the primitive variables (u, v, p) is used, where, the interpolation functions to represent the velocity and pressure are of the type P2-P1. To suppress the non-physical spatial oscillation that may occur in the numerical solution in the convection terms, the Streamline/Upwind Petrov Galerkin method is included to stabilize the Navier–Stokes equations. The temporal terms are discretized in an implicit fashion by using a second-order backward difference formula (BDF) scheme of the first and second order. The time steps are controlled by the numerical solver: adaptive time-stepping. The numerical accuracy was checked with a convergence study, where further mesh refinement and the decrease in the values of the relative tolerance (to lower the adaptive time step size in the solver) do not produce visible changes in the results so as to ensure mesh-independent solutions, as was done in previous CFD studies [25]. Figure 2a shows an example of a structure of the meshes used in the computations. The finest mesh size is provided adjacent to the pore wall to properly resolve the boundary layers, as shown in Figure 2b. Nine boundary layers were included. During the mesh refinement study, up to 15 boundary layers were considered. Variations in the rapid changes experienced by variables such as temperature, velocity and pressure in the wall normal directions were not observed during mesh refinement. Hence, it was considered that rapid changes in key variables are captured, appropriately including nine layers, because the increase in the number of boundary layers does not yield different solutions. No skewed elements were obtained in the boundary layer; however, in the roof boundary layer transition from quadrilateral to triangular elements, there are some regions where the mesh became anisotropic with some skewed elements. These skewed elements do not significantly affect the field of key variables, allowing a good transition from the quadrilateral elements to the triangular ones. For the as-processed microstructure, the number of elements is 94,350, whereas, for the largest scaling (60×) of the studied microstructure, the number of elements is 137,713. Convergence within each time step was tracked in the solver log where no failures were reported. The non-linear problem is solved by the damped Newton method with a constant value equal to 0.9 for the damping factor. The linearized subproblems that arise at each Newton iteration are solved by the direct solver PARDISO with the Nested dissection multi-threaded row pre-ordering. The studies were computed up to the time of 15 s, because, at this time, it was observed that the heat transfer process reaches a steady state in all cases.

### 2.4. Thermal Computational Homogenization

Macroscopically, the heterogeneous material can be assumed as a homogenous medium. The effective thermal conductivity ($k_{eff}$) may be calculated according to the Fourier law (Equation (15)), where the corresponding average quantities, such as heat flux, and temperature gradient (each represented as φ) can be obtained by taking a surface average using Equation (16).

(15) $$\bar{q_{y}}=k_{eff}\bar{\frac{\partial T}{\partial y}}$$

(16) $$\bar{\varphi }=\frac{1}{A_{REV}}\int_{A}\varphi dA_{REV}$$

The effective thermal conductivity is considered as additive contributions of the conductive ($k_{cd}$) and convective ($k_{cv}$) paths $k_{eff}=k_{cd}+k_{cv}$. More details on the numerical methodology for averaging quantities over the surface of the REV (2 D domain), used to predict material parameters (thermal conductivity) from constitutive equations (Fourier’s law) can be found in [10], which was also used to predict material parameters over volume microstructural analysis [26,27] using COMSOL Multiphysics.

## 3. Results and Discussion

The set of governing equations for the conjugate heat transfer–conduction in PVC foam structure, and conduction and convection in the air occluded in the cavities–were solved in an unsteady state in the as processed and scaled PVC microstructures, for which the equivalent diameters and individual cell are gathered in Table 3. During scaling, the shape factors remain constant, and only the size of the pores is increased.

### 3.1. Model Verification

**Figure 3:**
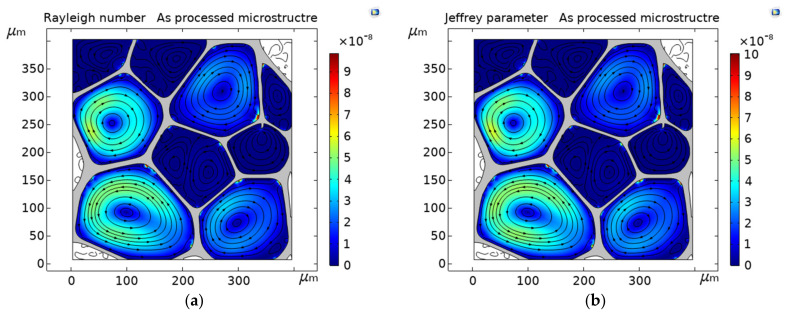
(**a**) Rayleigh number, and (**b**) Jeffreys parameter for the as procced PVC microstructure at time of 5 s. Streamlines represent vector velocity plotted at uniform density of 0.02. PVC (solid phase) is shown in gray color.

**Figure 4:**
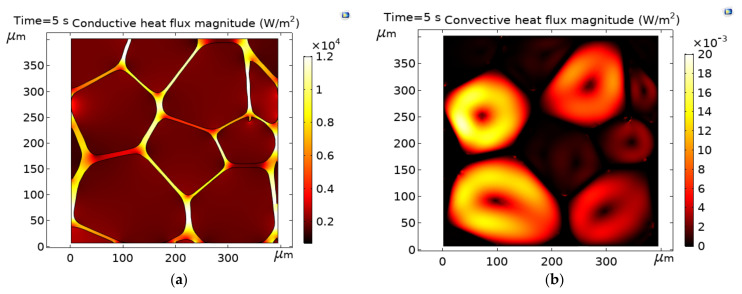
(**a**) Conductive heat flux magnitude, and (**b**) conductive heat flux magnitude for the procced PVC microstructure at time of 5 s.

The effective thermal conductivity in the Y-direction of the closed-cell PVC foam predicted in [6] is 0.036 ${\mathrm{Wm}}^{-1}\;K^{-1}$. In the present study, $k_{eff}$ is determined as a value of 0.0376 ${\mathrm{Wm}}^{-1}\;K^{-1}$. This result corresponds to the as-processed microstructure. At this average pore level of 0.121 mm, the Rayleigh number (Ra) (Figure 3a) and Jeffreys parameter (λ) (Figure 3b) have magnitudes in the order of 10^−8^, values out of range to have appreciable convective effects. For foam with 1 mm pores, it has been reported that the λ is four orders of magnitude lower than the critical value [11]. The results obtained here are in agreement with [11], and so it is expected that, as the pore size decreases, the dimensionless quantities Ra and λ are out of the range of convective effects. The conductive (Figure 4a) and convective (Figure 4b) heat fluxes have magnitudes in the order of 10^4^ and 10^−3^, respectively. Conductive heat transfer is about six orders of magnitude greater than the convective one, virtually eliminating the convective contribution to the effective thermal conductivity; therefore, gas convection is effectively inhibited at this pore size. In this case, the steady-state heat transfer was reached almost immediately at the time of 0.1 s. The numerical error was evaluated as $\left|\left(k_{reported}-k_{eff}\right)/k_{eported}\right|$, which gives a difference of lower than 5%. The computed results of $k_{eff}$ and the dimensionless quantities Ra and λ, are consistent with the data in [6,11], which proves the validity of the constructed model. According to these results, the model can accurately predict the thermal behavior of the PVC foam within the framework of conjugate heat transfer, and therefore, the subsequent studies are investigated effectively.

### 3.2. Temperature Distribution across the PVC Foam

**Figure 5:**
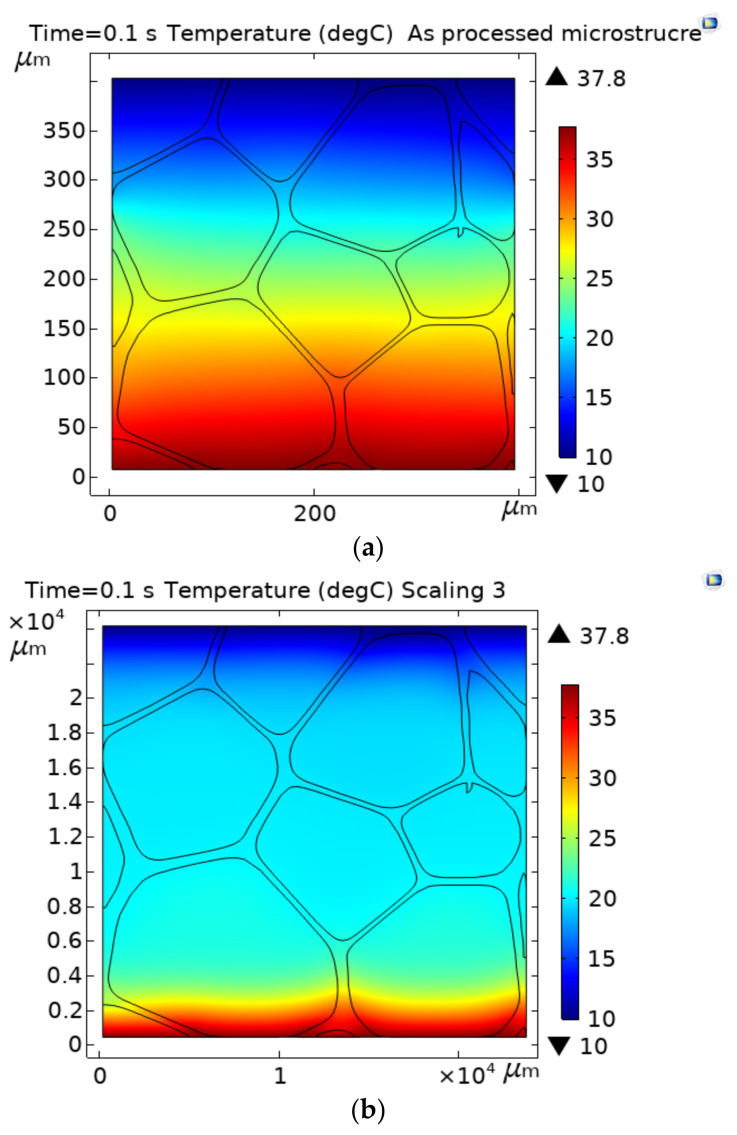

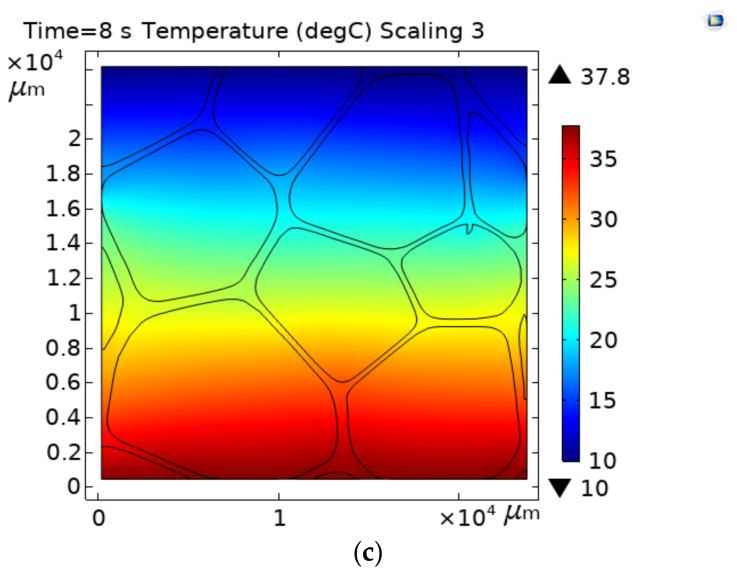
Temperature distribution in the processed microstructure and the scaling 3, measured at different simulation times.

Figure 5 shows the temperature distribution across the as-processed microstructure (Figure 5a), and the scaling 3 (60×), which is the higher scaling studied case (Figure 5b,c). These temperature distributions were obtained for a given differential temperature of 27.8 °C. In all cases, including scaling 1 (40×) and 2 (50×) (temperature distribution not shown), the temperature distributions across the material are similar at the steady-state. However, the time taken for the heat transfer process to reach steady-state was different in each case: for the processed microstructure, the time was 0.1 s, whereas, for scaling 1, scaling 2, and scaling 3, the times to reach a steady-state were, 3, 6, and 9 s, respectively. This is because, as the pore size is increased, the heat transfer distance is elongated, which, in turn, delays the heat flux, and modifies the temperature distribution across the material for a given time, before the steady heat transfer is stablished, as shown in Figure 5b,c. The increase in the heat transfer distance causes sharp temperature gradients across individual pores nearest to the hottest temperature, which progressively shift to neighboring cavities in the flow direction until to a steady-state is reached (Figure 5b). Sharp temperature gradients across the cavities, in combination with the pore sizes, which allow for the motion of fluid because of gas density variations, develop the convection currents. As the convective heat transfer is activated via temperature differences across individual cells; if the pore size allows the motion of air occluded, the thermal conductivity is temperature-dependent. These results are in agreement with reference [28], where increased heat flux depending on the variations in thermal conductivity for porous materials with low density were attributed to the creation of more convection and radiative heat mechanisms. On the other hand, it was observed that the convective heat transfer does not alter the temperature distribution across the material once the heat transfer reaches a steady-state, independently of the cavity sizes (Figure 5a,c). Therefore, the temperature distribution across the material in steady-state heat transfer is imposed by conduction, as this is the most dominant heat transfer mechanism.

### 3.3. Effective Thermal Conductivity ($k_{eff}$)

**Figure 6:**
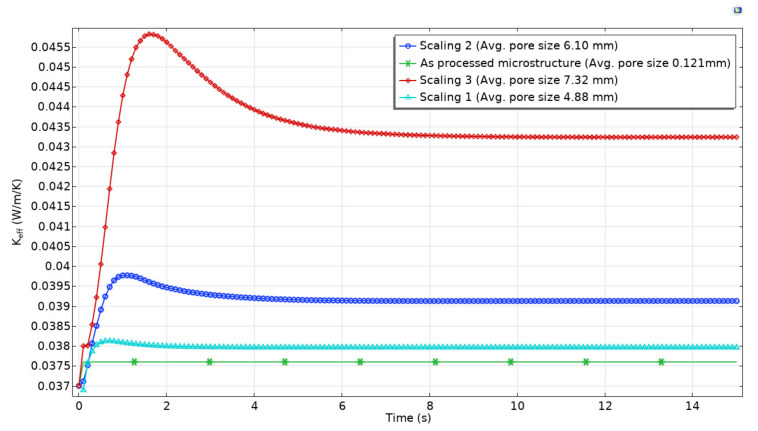
Variations in the effective thermal conductivity ($k_{eff}$) as function of cavity size and time.

Figure 6 shows the macroscopic response of the conjugate heat transfer analysis in the PVC foam microstructure as a function of time and average pore size. It is seen that, for the as-processed microstructure, which has an average pore size of 0.121 mm, the value of the effective thermal conductivity ($k_{eff}$) remains constant over time, with a value of 0.0376 ${\mathrm{Wm}}^{-1}\;K^{-1}$. For the scaling 1, which poses an average pore diameter of about 4.88 mm, the value of $k_{eff}$ is close to 0.038 ${\mathrm{Wm}}^{-1}\;K^{-1}$ at the time of 3 s. Prior to this time, the $k_{eff}$ value shows variations of marginal importance. For the scaling 2, which poses an average pore diameter of 6.10 mm, the steady-state heat transfer was reached at 6 s, and the value of $k_{eff}$ is about 0.0391 ${\mathrm{Wm}}^{-1}\;K^{-1}$. Before these 6 s, the thermal conductivity at this pore level showed significant variations. For the case of the scaling 3, which poses an average pore diameter of 7.32 mm, a constant value of 0.0433 ${\mathrm{Wm}}^{-1}\;K^{-1}$ was established at the time of 9 s. The maximum values of $k_{eff}$ reached during the unsteady heat transfer for the scaling 1, scaling 2, and scaling 3, were 0.0381, 0.0398, and 0.046 ${\mathrm{Wm}}^{-1}\;K^{-1}$, respectively. Concerning the constant value of 0.0376 ${\mathrm{Wm}}^{-1}\;K^{-1}$ produced by the as-processed microstructure, scaling 1, scaling 2, and scaling 3 showed an increase in the thermal conductivity value of 1.0, 4.0, and 13%, respectively. It can be seen that, for a given volume fraction of solid in the foam, the $k_{eff}$ increases as the pore size increases, both in steady and unsteady heat transfer.

Moreover, the time to reach steady heat transfer takes longer as cavity size increases, and the material thermal conductivity undergoes more variation. The maximum variations concerning the value of 0.0376 ${\mathrm{Wm}}^{-1}\;K^{-1}$ were found during the unsteady-state heat transfer, with differences of about 1.3, 5.8, and 22% for the scaling1, scaling 2, and scaling 3, respectively. Cellular materials with larger average pore sizes (∼6.10 mm) tent to promote easily unsteady-state heat transfer, and as a result, higher variations in the value of $k_{eff}$; therefore, its performance as an insulating material is affected by factors such as the operative temperature. On the contrary, the thermal performance of cellular materials with a small average pore size is less affected by the operative temperature, as long as the temperature does not fall below the condensation point of the air, since the liquid phase is more conductive than the gaseous phase, increasing heat transfer as reported in [28].

### 3.4. Onset of Convective Heat Transfer

**Figure 7:**
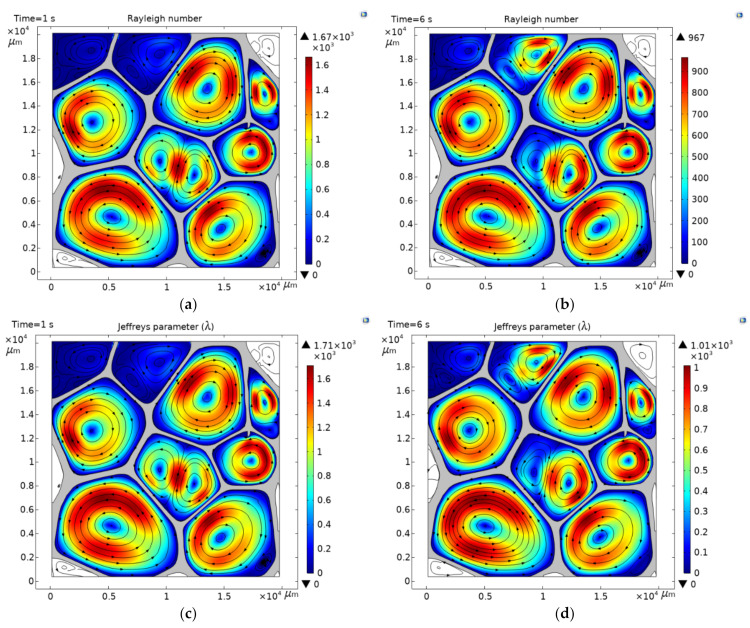
Higher Rayleigh number and Jeffreys parameter reached in unsteady (**a**,**c**)) and steady (**b**,**d**) state heat transfer for the scaling 2. Onset of convective heat transfer corresponds to an average pore size of 6.1 mm. Streamlines represent vector velocity plotted at uniform density of 0.02. PVC (solid phase) is shown in gray color.

Appreciable convective effects correspond to a Ra of 10^3^ or λ>1709. For the scaling 3, the average pore diameter is 7.32 mm. At this pore level, the dimensionless quantities are $Ra=3.38\times 10^{3}$, and $\lambda =3.54\times 10^{3}$ in steady-state heat transfer, while, in the unsteady-state $Ra=6.16\times 10^{3}$, and $\lambda =6.34\times 10^{3}$. The microscopic behavior of the interaction of the individual air convective currents within the cavities displays a macroscopic response of increased values of thermal conductivity, with respect a to constant value of up to 13% and 22% for the steady and unsteady state heat transfer, respectively (Section 3.3). In the case of the scaling 2, an increased thermal conductivity of 4.0% (steady-state heat transfer) and 5.8% (unsteady-state heat transfer) were found concerning the constant value (Section 3.3). Figure 7 shows the Rayleigh number and Jeffreys parameter high reached in unsteady-state heat transfer for the scaling 2 as $Ra=1.67\times 10^{3}$ and $\lambda =1.71\times 10^{3}$, at 1 s. In the steady-state heat transfer, these dimensionless quantities are Ra=967 and $\lambda =1.01\times 10^{3}$ at 6 s. A $Ra\approx 10^{3}$ indicates the onset of convective heat transfer within the foam. The value of $Ra\approx 10^{3}$(averaged in pore size) corresponds to the pore number one (which represents 21% of the pores), with a pore size of 9.06 mm, and it is circular in shape. It is seen that the criteria to consider natural convection are met only by cavity number one; however, convective currents are developed into several cells because of the thermal gradient stablished across the pores, although their sizes are lower than 9.06 mm, and they do not fulfill the criteria of $Ra\approx 10^{3}$ to consider natural convection within that cavities. Contrary to the case of open-celled foam, where convection took place in a global domain [19], in closed-cell foams, convection takes place in a local domain, but is not limited to cells that fulfill a $Ra\approx 10^{3}$ or λ>1709. Therefore, numerical calculations show that for air-based insulating materials the onset of convective heat transfer corresponds to an average pore size of 6.1 mm independently if heat transfer is in a steady or unsteady state. Below this pore level, convection is suppressed because conduction heat transfer within the air reduces the temperature gradient across the fluid or the stratifications in temperature, which lead to density variations. On the other hand, the contribution to conductivity by convection produces an increment of about 4.5% in the value of $k_{eff}$.

### 3.5. Conductive and Convective Heat Flux Magnitudes

**Figure 8:**
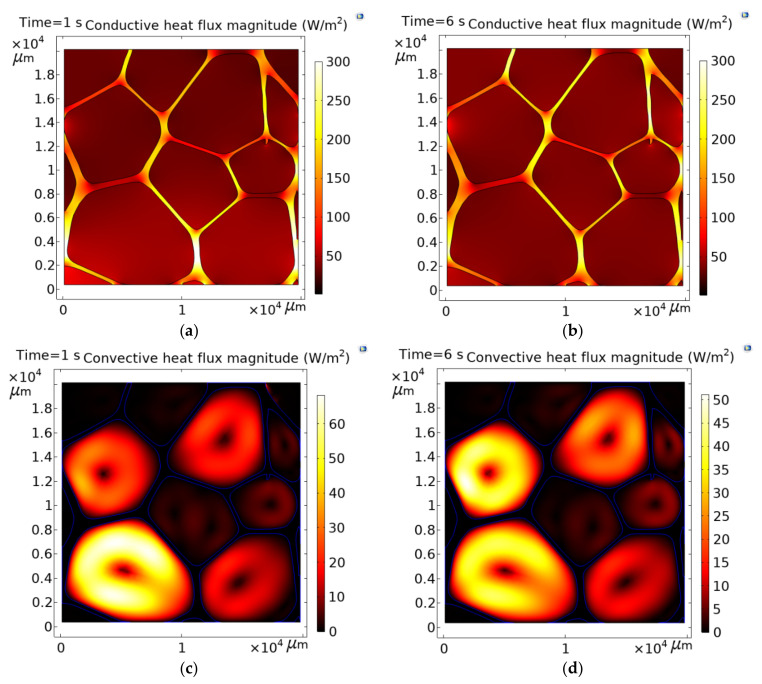
Amount of heat transported by convection and conduction within the foam for the unsteady (**a**,**c**) and steady (**b**,**d**) states.

Figure 8 shows the conductive and convective heat flux magnitudes during unsteady and steady-state heat transfer for the scaling 2. A comparison between the heat flux shown in Figure 4a, where convection is virtually eliminated by conduction, and Figure 8 a,b, shows that the conduction paths are not altered by the creation of convection flux in unsteady or steady-state heat transfer. In addition, the air circulations (streamlines) within the cavities keep the same directions in all cases, indicating that the heat is gained in the air from the same point of the wall. Figure 7c,d also shows that variations in the size and shape of the cavities produce different behavior of the convective currents, but in the central core of the currents, convection is suppressed because, in the core, the fluid is stagnant. For pores that exhibit notable changes in the aspect ratio, for example, the square and ellipsoidal shapes, such as the pore numbers 4 and 7, (see Figure 1 for the numbering of pores) two circulation currents are formed. These results support the fact that convective transport is anisotropic in foams, as documented in [11]. From the results shown in Section 3.3, Section 3.4 and Section 3.5, it can be generalized that the amount of heat transported by convection is about 20% of the heat transported by conduction within the foam for the onset of convective heat transfer, which produces an increasing average of the $k_{eff}$ of about 4.5% with respect to a constant value. A study suggests that radiation may contribute 6–26% of the effective conductivity [5]. According to these results, conduction is the most dominant energy transfer mechanism in cellular materials, followed by radiation and convection.

## 4. Conclusions

This work extends the field of study of the convective heat transfer mode in air-filled cellular structures, a mechanism generally ignored in the heat transfer studies of these systems. The main results to be highlighted are as follows.

For a given volume fraction, as the pore size is increased, the heat transfer distance is elongated, delaying the heat flux. As a result of this, sharp temperature gradients across individual pores nearest to the hottest temperature arises, these temperature gradients progressively shift to neighboring cavities in the heat flow direction until a steady-state is reached. Sharp temperature gradients across the cavities, combined with the cavity sizes that allow the motion of fluid because of gas density variations, activate the creation of convection. Due to the convection heat transfer is activated via temperature differences across individual cells, this effect causes that the thermal conductivity of porous materials with low density to be temperature-dependent. The time taken to reach a steady-heat transfer takes longer as the cavity size increases, which causes the material thermal conductivity to undergo more variations; then, the performance of porous materials with low density as an insulating material is affected by factors such as the operative temperature. For air-based insulating materials, the onset of convective heat transfer corresponds to an average pore size of 6.1 mm independently of whether heat transfer is in a steady or unsteady state. Hence, the maximum average pore size found to suppress the convective heat transfer is 6.0 mm. The amount of heat transported by convection is about 20% of heat transported by conduction within the foam for the onset of convective heat transfer, which, in turn, produces an increasing average of the $k_{eff}$ of about 4.5% respect to a constant value. In closed-cell foams, convection takes place in a local domain, but it is not limited to cells that fulfill a $Ra\approx 10^{3}$ or λ>1709 because the convective cells interact with the neighboring cavities.

## Figures and Tables

**Table 1:** Shape factor of regular pore shape [24].

Pore Shape	Shape Factor	Pore Shape	Shape Factor
Roundness	1.00	Ellipse (1:4)	1.89
Square	1.27	Rectangle (1:2)	1.43
Ellipse (1:2)	1.19	Rectangle (1:3)	1.69
Ellipse (1:3)	1.51	Rectangle (1:4)	2.29

**Table 2:** The equivalent diameters, and the shape factors of individual cell in the processed image of PVC foam.

Pore Number	Equivalent Diameter (mm)	Shape Factor	Pore Shape
1	0.181	1.08	Circular
2	0.151	1.08	Circular
3	0.091	1.16	Ellipse (1:2)
4	0.127	1.11	Ellipse (1:2)
5	0.150	1.06	Circular
6	0.150	1.12	Ellipse (1:2)
7	0.098	1.26	Square
8	0.081	1.39	Circular *
9	0.064	1.43	Rectangle (1:2)

* The shape factor obtained for pore number eight is close to a rectangle (1:2); however, as symmetry is considered in the boundaries of the REV, actually the shape of this cavity is similar to the shape of pore number five, which is close to a circle.

**Table 3:** The equivalent diameters of individual cells in the as processed, and scaled size of the microstructure of closed-cell PVC foam.

	Equivalent Diameter (mm)			
Pore Number	As Processed Microstructure	Scaling 1 (40×)	Scaling 2 (50×)	Scaling 3 (60×)
1	0.181	7.25	9.06	10.9
2	0.151	6.08	7.6	9.11
3	0.091	3.66	4.58	5.49
4	0.127	5.1	6.37	7.65
5	0.150	6.03	7.54	9.04
6	0.150	6.02	7.53	9.04
7	0.098	3.95	4.94	5.93
8	0.081	3.25	4.07	4.88
9	0.064	2.59	3.24	3.88
Avg.	0.121	4.88	6.10	7.32

## Data Availability

The data presented in this study are available on request from the corresponding author.
